# Anemia of Inflammation Is Related to Cognitive Impairment among Children in Leyte, The Philippines

**DOI:** 10.1371/journal.pntd.0000533

**Published:** 2009-10-20

**Authors:** Courtney L. Olson, Luz P. Acosta, Natasha S. Hochberg, Remigio M. Olveda, Mario Jiz, Stephen T. McGarvey, Jonathan D. Kurtis, David C. Bellinger, Jennifer F. Friedman

**Affiliations:** 1 Center for International Health Research, Rhode Island Hospital, Providence, Rhode Island, United States of America; 2 Department of Pediatrics, Alpert Medical School of Brown University, Providence, Rhode Island, United States of America; 3 Research Institute for Tropical Medicine, Manila, The Philippines; 4 Emory University Hospital, Department of Medicine, Atlanta, Georgia, United States of America; 5 Epidemiology Section, Department of Community Health and International Health Institute, Brown University, Providence, Rhode Island, United States of America; 6 Department of Pathology and Laboratory Medicine, Alpert Medical School of Brown University, Providence, Rhode Island, United States of America; 7 Department of Environmental Health, Harvard School of Public Health Boston, Massachusetts, United States of America; 8 Department of Neurology, Children's Hospital, Boston, Massachusetts, United States of America; Case Western Reserve University School of Medicine, United States of America

## Abstract

**Background:**

Many studies have addressed the relationship between iron deficiency anemia (IDA) and cognitive impairment, but none have evaluated the role of non-iron deficiency anemia (NIDA). One of the main causes of NIDA in developing countries is AI, largely due to infectious diseases, whereby iron is shunted away from bio-available forms to storage forms, making it less accessible for use by host tissues. The objective of this study was to determine the effect of NIDA, due largely to AI in this context, on cognitive function after adjustment for potential confounders.

**Methodology:**

This cross-sectional study was conducted in Leyte, The Philippines among 322 children ages 7–18 years. Blood samples were collected and analyzed at the time of cognition testing. Three stool samples were collected and evaluated by the Kato Katz method for quantitative assessment for *Schistosoma japonicum* and geo-helminth infection. Socio-economic status (SES) was evaluated by survey. Linear regression models were used to quantify the adjusted relationship between performance in different cognitive domains and both IDA and NIDA.

**Principal Findings:**

After adjusting for age, sex, SES and nutritional status, children in the NIDA had lower scores on the PNIT (*P* = <0.05) and the WRAML memory domain (*P*<0.05) compared to children in the non-anemic group. Children in the IDA had lower performance on the PNIT compared to the non-anemic group after controlling for potential confounders (*P*<0.05).

**Conclusions:**

NIDA, predominantly due to AI in this context, was related to lower performance on two tests of cognitive function. This is likely due to decreased delivery of iron to host tissues in this context, including the CNS.

## Author Summary

Past studies have demonstrated that iron deficiency anemia is related to deficits in cognitive fucntioning in children, and treating iron deficiency anemia with iron supplementation can improve cognition. Anemia of inflammation is another type of anemia caused by many diseases of lesser-developed countries including bacterial and parasitic infections. Anemia of inflammation is characterized by disordered iron metabolism, such that iron is sequestered in storage forms, preventing its use from tissues that require it. We hypothesized that decreased iron delivery to the brain in the context of anemia of inflammation might lead to decreased cognitive performance. This study found that children with anemia of inflammation had decreased cognitive performance in specific domains, compared to subjects with no anemia. True total body iron deficiency anemia was related to lower performance in the same domains. The only treatment option for anemia of inflammation is treatment of the underlying disease. Iron supplementation will not prevent cognitive deficits in children with anemia of inflammation. Interventions aimed towards maximizing the cognitive development of children in lesser-developed countries will need to focus on the prevention and treatment of bacterial and parasitic infections.

## Introduction

Anemia of inflammation (AI), is the second most prevalent type of anemia following iron deficiency anemia (IDA) [1],[2]. The most common conditions associated with AI include bacterial, viral, and parasitic infections, cancers, and autoimmune diseases [1]. AI leads to alterations in iron metabolism likely mediated by elevated levels of specific cytokines in response to the aforementioned disease processes. Hepcidin, an acute phase protein induced by interleukin-6, has been proposed as the link between inflammatory disorders and AI [3],[4]. Its actions include both blocking absorption of iron from the gut and its release from reticuloendotheial macrophages. In previous work in this study population we have identified AI as the predominant cause of S. *japonicum*-related anemia [5],[6].

Children and adolescents with IDA perform less well on specific cognitive tests than those without IDA [7]. Further, treatment of IDA has been demonstrated to improve cognitive performance among children and adults [8],[9],[10]. The cognitive deficits seem to be mediated by low iron levels as opposed to anemia itself. For example, a randomized controlled trial conducted among non-anemic iron deficient adolescent females demonstrated improved verbal learning and memory with iron supplementation [11], suggesting cognitive deficits are most related to decreased delivery of iron to the central nervous system (CNS).

In the context of AI, the serum concentration of iron and the iron saturation of transferrin are also decreased leading to decreased iron delivery to host tissues that necessitate it [1]. One well known consequence is iron deficient erythropoiesis, whereby anemia results despite normal total body supplies of iron, as iron is shunted away from bio-available forms, into storage forms. We hypothesized that AI might be associated with decreased cognitive function in domains sensitive to CNS iron status, due to decreased bio-availability of iron. Iron is required for many essential brain functions including myelination and synthesis of the neurotransmitters serotonin and dopamine [7]. It is also possible, as demonstrated in recent studies, that pro-inflammatory cytokines made in the context of parasitic diseases, which cause AI, might have direct effects on cognitive processing [12]. No studies have assessed the relationship between AI, pro-inflammatory cytokines elaborated in this context, and cognitive function in children.

The main objective of this study was to assess the relationship between NIDA, largely due to AI in our population, and cognitive function after adjustment for potential confounding factors. The secondary objectives were to 1) assess the relationship between IDA and cognitive domains not previously studied, with more careful control for confounders and 2) explore mechanisms through which helminth infections could mediate cognitive impairment through established relationships with both AI [5],[6] and pro-inflammatory cytokines [13],[14],[15]. We hypothesized that anemia of inflammation would lead to lower cognitive performance in domains sensitive to brain iron content and that this would be more important than serum levels of pro-inflammatory cytokines, which have been demonstrated to lead to impaired cognitive performance.

## Methods

### Study population

This study was conducted in Macanip, a rural rice farming village in Leyte, The Philippines, where *S. japonicum* and geo-helminth infections are prevalent. There is no malaria in this study area. Three to six months before the start of the study, a census was completed for the entire village. Study staff went door to door accompanied by a resident of the village, mapping each household with global positing satellite devices. The census enumerated number of individuals in the household, their gender, and dates of birth. At that time, all individuals in the household ages 8–30 were asked to provide informed consent for the stool screening procedures for a study investigating immune correlates of re-infection. The latter, longitudinal study only enrolled infected subjects. For this cross-sectional study, we used the initial census data to a) identify infected subjects ages 7–18 who were eligible for the longitudinal study and b) identify uninfected subjects ages 7–18 who were only eligible for this cross-sectional study. Exclusion criteria included pregnancy or lactation or the presence of a serious chronic disease determined by history, physical examination, or laboratory findings. An attempt was made to recruit all age eligible subjects (N = 394), however, 14 could not be re-located or scheduled for assessments, leaving 380 enrolled subjects. A separate consent process was used for the activities related to this study, which included the cognition testing for all subjects and a single blood test for the uninfected subjects, who otherwise would not have had this blood sample taken as part of their participation in the longitudinal study. Cognition testing occurred between 9/7/2002–10/8/2002 for all subjects. Treatment for infected subjects as well as the blood sample and all other assessments were taken between 10/28/02–11/6/02.

For all study activities, written informed consent was obtained from the parents of all study subjects and assent was provided by all children over the age of 8 who could understand the assent form and process. The study was approved by the Brown University and Philippines Research Institute of Tropical Medicine Institutional Review Boards.

### Cognitive tests

The choice of cognitive tests employed was based on 1) known relationships between iron status and both memory (WRAML verbal memory and verbal fluency) and concept recognition (PNIT) domains [16],[17] and 2) known relationships between helminth infections and cognitive domains (verbal fluency and WRAML Learning) [18],[19].

The cognitive tests were translated and adapted for use in The Philippines. They were pilot tested among Filipino children (N = 51) from other villages near the study area. Reliability testing for each cognitive test included joint inter-rater and test-retest reliability with a six-week interval between tests. Cronbach's alpha coefficient was used to assess to the degree of internal consistency among the three subscales that constitute each of the learning and memory domains of the Wide Range Assessment of Memory and Learning (WRAML).

#### Wide range assessment of memory and learning

The WRAML was chosen so that we could address the effects of anemia on learning processes, about which little is known in developing countries. The WRAML was developed in the United States (US) and was age standardized to a nationally representative sample of more than 2,300 children 5–17 years old [20]. Specific subtests of the WRAML have been validated against school performance and have been shown to be significantly related to academic achievement in particular subject areas. We used two of the three cognitive indices, verbal memory and learning, as the third index was too difficult to adapt. Age-standardized scores were created for both the verbal memory and learning indices.

#### Verbal fluency

The verbal fluency test has previously been described as an acceptable task across a wide range of cultures [21]. A child is asked to name as many items in a given category in 60 seconds after a practice category is given. Verbal fluency is considered a good indicator of the central executive component of working memory [19],[21].

#### Philippines non-verbal intelligence test (PNIT)

This test was developed in The Philippines by American and Filipino psychologists [17]. The PNIT measures concept recognition and abstract thinking. It involves 100 cards that each depict five items, one of which does not fit the concept portrayed. The test had found excellent reliability characteristics among rural Filipino children (rho = 0.71–0.95) [17]. In addition, the validity of this test was also compared with the grades that children had received in related school classes and was found to have validity coefficients very similar to those found with cognitive tests among American children.

### Blood collection, processing, and analysis

A complete blood count was obtained using a hematology analyzer (Serono Baker Diagnostics) on venous blood samples. Serum ferritin (SF), serum transferrin receptor (sTfR), C-reactive protein (CRP), and serum cytokines (interleukin-6 (IL-6), interferon gamma (IFN-γ), tumor necrosis factor alpha (TNF-α) were analyzed using a multiplex bead-based platform (BioRad Hercules, CA) as described previously [14]. SF is a measure of stored iron. sTfR is a circulating form of the transferrin protein receptor, derived mostly from red blood cell precursors. sTfR is increased when red blood cells are iron deficient, as the receptor is upregulated to increase iron uptake, and with expanded red blood cell in under various clinical settings [22].

### Stool examination

Parasite egg counts were determined by examination of consecutive stool specimens obtained from each study participant on separate days. We requested three stool specimens from each subject, but individuals were eligible if they provided one or two specimens. All subjects provided at least one sample and 82.8% and 64.8% provided two or three samples, respectively. Each stool specimen was evaluated in duplicate for *S. japonicum*, *Ascaris lumbricoides*, *Trichuris trichiura*, and hookworm eggs by the Kato Katz method within 24 hours of collection. The mean eggs per gram of stool was used as the quantitative measure of infection status for each worm based on the average egg count for each duplicate specimen. Then, the mean egg count was taken across the stool samples (1–3), including samples with zero epg. World Health Organization criteria were used to classify each infection as uninfected or low, moderate, or high intensity [23].

### Mediating/confounding covariates

#### Socioeconomic status

SES was based on data from a 47 item questionnaire addressing parental and child educational status, occupation, sanitation, home and land ownership, and assets. The child's education status addressed whether the child was currently enrolled in school and whether or not the child ever missed a year of school. Highest grade completed by both parents and the head of household was ascertained. Questions related to household economic status included a measure of crowding, materials from which the home was constructed, and assets. Three categories of occupation were created that captured farming, unskilled, and skilled labor. The questionnaire had good internal consistency (Cronbach's alpha of 0.82). A composite SES score using all questionnaire items was determined using principal components analysis to appropriately weight items [24]. It was not possible to calculate a summary SES score for 49 subjects due to missing data. For these subjects, SES scores were imputed from scores of another person in the same household or from the overall mean score of persons of the same age and sex if a household value was not available.

#### Nutritional status

Subjects were weighed to the nearest 0.1 kg on a Seca Model 880 Digital scale (Hanover, MD), and height was measured to the nearest 0.1 cm by use of a portable anthropometer [25]. These measurements were used to determine the height-for-age z-score (HAZ), body mass index (BMI) [wt/ht^2^] and BMI z-score (BMIZ). Center for Disease Control reference curves were used to calculate these z-scores by use of EpiInfo software (version 2000, Atlanta, Georgia).

#### Pubertal developmental stage

Tanner staging of pubertal development was performed by two trained physicians according to standard criteria [26]. Tanner staging provides an estimate of an individual's current pubertal developmental status. Tanner stage I denotes pre-pubertal status and Tanner stage V denotes completion of pubertal development.

### Definitions

Anemia was defined on the basis of age- and sex-specific hemoglobin cut-off values recommended by the WHO: hemoglobin <11.5 g/dL for children aged <12 years, hemoglobin <12.0 g/dL for males aged 12–14 years and females ≥12 years, and hemoglobin <13.0 g/dL for males aged ≥15 years [27]. Iron deficiency anemia (IDA) was defined as the presence of anemia and serum ferritin (SF) <12 ng/ml in children younger than 15 years and females of all ages, and SF<18 ng/ml in males age 15 years and older. Non-iron deficiency anemia (NIDA) was defined as the presence of anemia and SF≥12 ng/ml in children younger than 15 years and females of all ages and SF≥18 ng/ml in males age 15 years and older. SF best demonstrates reticuloendothelial iron status [2]. A limitation of using SF to determine iron status is its “false” elevation in the context of acute inflammation [28]. For this reason, we used an alternative SF cutoff of 30 ng/ml to define iron deficiency in individuals with concurrent inflammation (CRP level of >8.2 µg/ml) [27]. This definition is conservative in that individuals with ongoing inflammation and iron deficiency are less likely to be misclassified as iron-replete.

In addition, we sought to rule out other causes of NIDA. To this end, we evaluated mean corpuscular volume (MCV) with a cut-off of 100 fL to define macrocytosis. We defined the presence of hyperbilirubinemia, a marker for hemolysis, as indirect bilirubin >1 mg/dL. Splenomegaly was defined as spleen size >2 standard deviations (SD) above the reference mean of a healthy Chinese population [29].

### Statistical analyses

Separate linear regression models were made for each cognitive test to quantify the relationship between performance on cognitive tests and both NIDA and IDA. Children with no anemia were the reference group for all analyses. Analyses were done in SAS software version 9.0 (SAS Institute, Cary, NC). Variables were evaluated as confounders of the relationship between anemia type and cognitive ability if they were independently associated with both anemia and cognitive performance in bivariate analyses. Potential confounders included sex, age, SES, and nutritional status. In our previous work, we found that specific helminth infections were related to cognitive deficits [18], and to AI [5],[6] therefore, the presence of these infections were added to final models for two reasons: 1) to assess whether helminth infection status confounded the relationship between cognition and anemia type such that the true independent predictor was helminth infection and 2) to assess the mechanistic role of AI in mediating the relationship between helminth infections and cognitive impairment. We evaluated whether the beta coefficient for the anemia status covariate changed by more than 10%, suggesting either confounding or inclusion of two variables in a causal pathway. Finally, given pro-inflammatory cytokines are related to both specific deficits in cognitive functioning and NIDA, we evaluated the direct effect of TNF-α, IFN-γ and IL-6 on cognitive outcomes [12],[30],[31], without anemia status in the model.

A significant proportion of the variance of our outcome measures was attributable to clustering within household. Therefore, multi-level statistical analyses were used to adjust for clustering at the household level. Specifically, multivariate random intercept models were implemented using Proc Mixed(with household as random effect and a compound symmetry correlation matrix. Least square (LS) mean values represent the mean adjusted for confounders in multivariable models.

## Results

Of the 380 children enrolled in the study, 44 did not complete cognition testing and 14 subjects had incomplete data with respect to anemia status, leaving an effective study sample of 322 children.

Table 1 presents the results of the reliability characteristics for the four cognitive tests. Inter-rater reliability (IRR) tests conducted among 51 subjects ages 8–16 demonstrated good consistency between raters of children's test scores (IRR≥0.98 for all tests). The test-retest analyses among 36 subjects showed good to excellent reliability (0.61–0.89). The degree of consistency among the subscales of the WRAML was fair-good. In addition, we assessed the validity of the tests in our study sample by comparing cognitive test scores to known factors related to cognitive performance such socio-economic status and found significant correlations (rho = 0.216–0.293, all P<0.001).

**Table 1:** Cognitive test reliability.

Cognitive Test	Inter-rater (joint reliability)	Test-retest	Cronbach alpha‡
WRAML Verbal Memory index	0.99	0.7	0.81
WRAML Learning index	0.99	0.79	0.54
PNIT	0.99	0.89	N/A
Verbal fluency	0.98	0.61	N/A

WRAML = Wide Range Assessment of Memory and Learning; PNIT = Philippine Non-verbal Intelligence Test; NA = not applicable.

**‡:** Cronbach's alpha, used for correlation among three subscales that constitute each WRAML subtest.

We first evaluated the role of other potential causes of NIDA (Table 2). We found that no subjects in the NIDA group were macrocytic, making deficiency of folate and vitamin B12 unlikely causes of anemia. In the NIDA group, only one subject had elevated indirect bilirubin (1.07 mg/dL) and one subject had splenomegaly.

**Table 2:** Characteristics of the study sample pooled and across anemia types.

Variable	Entire Cohort	IDA	NIDA	No Anemia
	*n* = 322 (100%)	*n* = 57 (17.7%)	*n* = 56 (17.4%)	*n* = 209 (64.9%)
Age (y)^1^	12.1 (11.7, 12.4)	12.1 (11.3, 12.8)	11.3 (10.6, 12.1)^*^	12.2 (11.8, 12.7)
Males [*n*(%)]	179 (55.6%)	44 (77.2%)^***^	32 (57.1%)	103 (49.3%)
Socioeconomic status score^1^^,^ 2	2.33 (2.23, 2.43)	1.89 (1.67, 2.11)^***^	2.24 (2.03, 2.44)	2.47 (2.34, 2.60)
Hemoglobin (g/dL)^1^	12.0 (11.8, 12.2)	9.7 (9.3, 10.2)^***^	10.8 (10.5, 11.0)^***^	13.0 (12.8, 13.1)
Serum ferritin (ng/mL)^1^	37.3 (33.0, 41.6)	9.5 (7.7, 11.3)^***^	52.2 (39.2, 65.2)	41.0 (35.8, 46.2)
sTfR (mg/L)^1^	7.0 (6.0, 8.0)	8.8 (7.6, 10.1)^**^	7.9 (5.7, 10.1)	6.3 (4.9, 7.7)
CRP (µg/mL)^1^	11.6 (8.7, 14.5)	17.5 (11.4, 23.5)^**^	23.8 (12.1, 35.5)^**^	6.6 (4.2, 9.1)
BMI Z-score^1^	−1.2 (−1.3, −1.0)	−1.2 (−1.5, −1.0)	−1.3 (−1.5, −1.1)	−1.1 (−1.2, −0.9)
*Schistosoma japonicum* [*n* (%)]	251 (78.0%)	50 (87.7%)^***^	53 (94.6%)^***^	148 (70.8%)
Hookworm [*n* (%)]	149 (47.3%)^3^	31 (54.4%)^*^	31 (56.4%)^4^^ *^	87 (42.9%)^5^
*Ascaris lumbricoides* [*n* (%)]	240 (76.2%)^3^	40 (70.2%)	46 (83.6%)^4^	154 (75.9%)^5^
*Trichuris trichiura* [*n* (%)]	298 (94.6%)^3^	55 (96.5%)	52 (94.5%)^4^	191 (94.1%)^5^
Macrocytosis (MCV>100 fL)	0 (0%)	0 (0%)	0 (0%)	0 (0%)
Hyperbilirubinemia^6^	17 (5.3%)	1 (1.8%)	1 (1.8%)	14 (6.8%)
Splenomegaly^7^	5 (1.6%)	4 (7.0%)	1 (1.8%)	0 (0%)
WRAML Verbal Memory Index^1^	65.4 (64.1, 66.8)	61.7 (58.8, 64.6)^**^	63.0 (60.6, 65.5)^**^	67.3 (65.5, 69.0)
WRAML Learning Index^1^	87.8 (86.1, 89.5)	83.5 (79.1, 87.8)^*^	86.7 (82.8, 90.6)	89.5 (87.3, 91.6)
PNIT^1^	28.4 (27.6, 29.2)	26.3 (24.0, 28.6)^**^	26.3 (24.5, 28.0)^**^	29.7 (28.7, 30.7)
Verbal Fluency	18.0 (17.4, 18.6)	17.0 (15.6,18.5)	17.3 (16.2, 18.5)	18.6 (17.9, 19.3)

IDA = iron deficiency anemia; NIDA = non-iron deficiency anemia; sTfR = soluble transferrin receptor; CRP = C-reactive protein; BMI = body mass index; WRAML = Wide Range Assessment of Memory and Learning.

^*^P<0.05, **P<0.01, ***P<0.001 compared to the No Anemia group.

1 *x*−; 95% CI in parentheses (all such values except for otherwise indicated).

2 Summary score of all questionnaire items calculated by principal components analysis.

3 *n* = 315.

4 *n* = 55.

5 *n* = 203.

6 Indirect (unconjugated) bilirubin >1 mg/dL.

7 Spleen size >2 standard deviations (SD) above the reference mean of a healthy Chinese population.

We then evaluated our definitions of IDA and NIDA and found these were supported by sTfR relationships among our study subjects. (Table 2) Levels of sTfR in AI are not significantly different from sTfR levels in those with no anemia, as sTfR expression is downregulated by inflammatory cytokines [1]. In our study sample, the IDA group had the highest mean level of sTfR compared to both NIDA and non-anemic (*P*<0.01).

Other characteristics of the study population at baseline and by anemia status are presented in Table 2. The prevalence of NIDA was 17.4%, and the prevalence of IDA was 17.7%. Children with IDA were significantly more likely to have a low SES compared to those with no anemia (*P*<0.001). Children with IDA or NIDA had significantly higher prevalences of *S. japonicum* and hookworm infections compared with non-anemic children.

**Figure 1:**
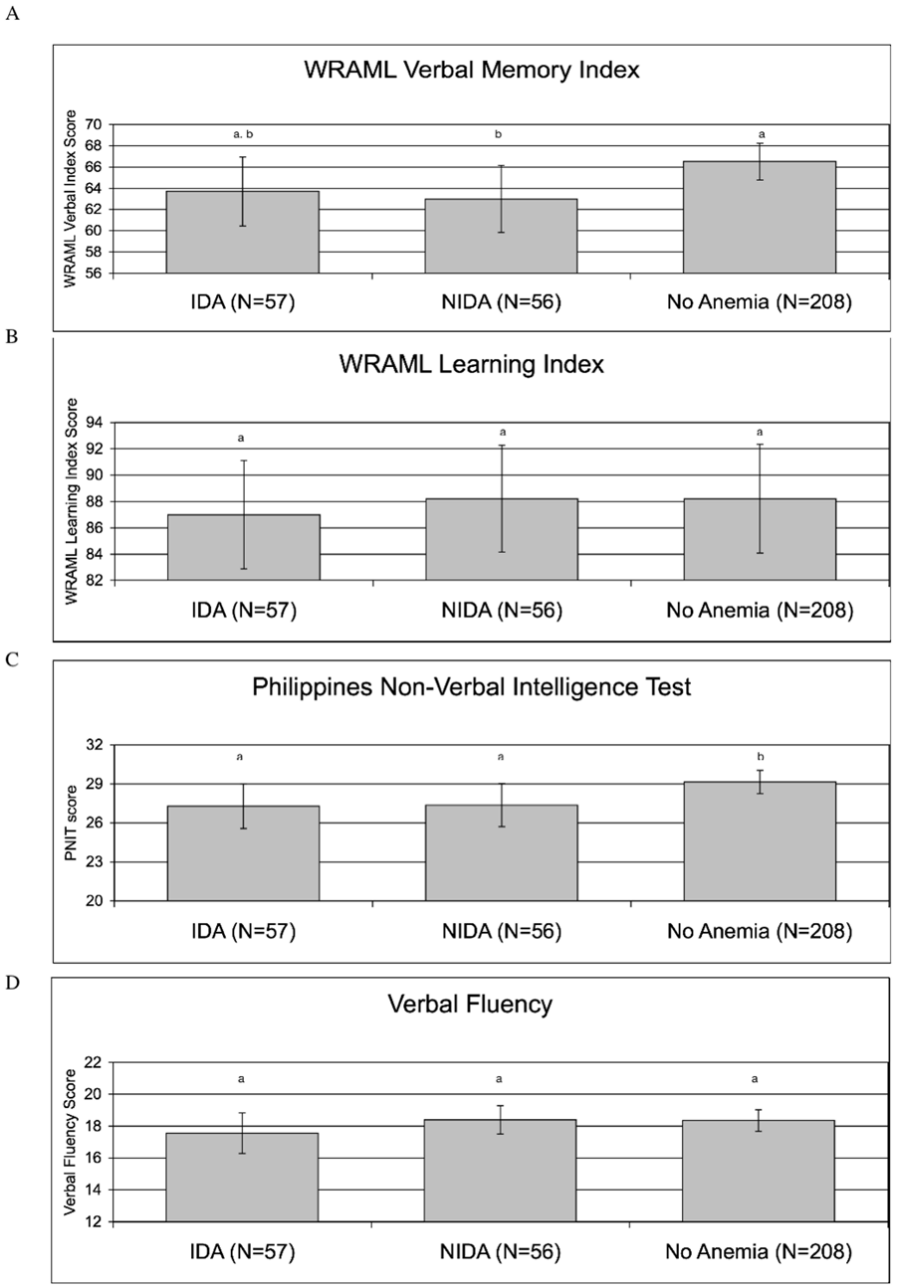
Least Squares Mean Scores on Tests of Cognition by anemia category after adjustment for age, sex, socio-economic status, and body mass index z-score. Error bars represent 95% confidence intervals. ^a, b^ Different letters represent significant differences (P<0.05) in scores across anemia categories. IDA = Iron Deficiency Anemia; NIDA = Non-Iron Deficiency Anemia; WRAML = Wide Range Assessment of Memory and Learning.

Figure 1 shows the mean cognitive scores across anemia groups adjusted for age, sex, SES, and BMI z-score.

### 

#### Verbal memory index of WRAML

Children with NIDA performed significantly worse on this test compared to children with no anemia after adjusting for confounding variables (*P*<0.05). Children with IDA scored 2.8 corrected points lower than children with no anemia, but this did not reach statistical significance (*P* = 0.12). The other covariates significantly associated with lower performance on the verbal memory index included, SES, nutritional status, and sex, with boys performing worse than girls.

#### Learning index of WRAML

There were no significant differences among children with either IDA or NIDA in performance on this test compared to those with no anemia. However, SES and sex were related to performance on the learning index, with boys performing worse than girls.

#### PNIT

Both IDA and NIDA were significantly associated with lower scores on the PNIT compared to the no anemia group after adjusting for confounding variables (*P*<0.05). Other predictors of lower performance on the PNIT, which is not age or sex standardized, included age, sex, and SES.

#### Verbal fluency

Anemia, regardless of type, was not associated with decreased performance on the verbal fluency test compared to the non-anemic group, after confounder adjustment. Age and SES were significantly and positively related to children's performance on this test, which is not age standardized.

In addition we investigated interaction effects to assess whether age or developmental status modified the relationship between anemia group and cognitive performance. Given developmental stage is likely a more important determinant of cognitive processing than age, we examined the interaction between anemia group and Tanner stage dichotomized as Tanner stage 1 or 2 or Tanner stage >2. None of the interaction terms were significant for any of the cognitive tests.

To explore the mechanistic role of helminths in mediating the relationship between NIDA and cognitive impairment, we evaluated multivariate models controlling for age, SES, sex, and nutritional status with and without helminth egg counts. Previous studies in this population demonstrated an association between *A. lumbricoides* and poorer performance on the verbal memory index of WRAML [18]. The relationship between NIDA and cognitive performance on the verbal memory index of the WRAML was only slightly attenuated (<10%), however, by controlling for *A. lumbricoides* in the model, suggesting this is not a primary mechanism through which *A. lumbricoides* causes cognitive impairment and was not confounding this relationship. In our prior work, S. *japonicum* was related to lower scores on the WRAML learning index and T. *trichurius* to verbal fluency, however, since NIDA was not related to lower performance in either of these cognitive tests, no further analyses were conducted.

We examined the independent effect of TNF-α, IFN-γ and IL-6 on cognitive tests by removing anemia status from the model and including only one cytokine and the aforementioned confounders (Table 3). We found that none of these three cytokines were significantly related as main effects to performance on any test. Given these cytokines were not independently related to cognitive outcomes in this setting, they are unlikely to be primary determinants in a causal pathway linking AI to cognitive impairment. Therefore, no further analyses were conducted.

**Table 3:** Multivariate models assessing the adjusted association between cognition test scores and pro-inflammatory cytokines.

Variable	WRAML^‡^ Verbal Memory Index	WRAML Learning Index	PNIT	Verbal Fluency
	β coefficient	β coefficient	β coefficient	β coefficient
	(P value)	(P value)	(P value)	(P value)
IL-6	0.19 (0.37)	0.26 (0.31)	0.03 (0.75)	−0.08 (0.28)
Age	−0.19 (0.44)	−0.08 (0.81)	1.17 (<0.001)	0.67 (<0.001)
Sex (Male)	−2.95 (0.04)	−7.19 (<0.001)	−2.41 (0.001	−1.18 (0.03)
SES	2.40 (0.01)	5.15 (<0.001)	1.77 (<0.001)	0.95 (<0.01)
BMI Z-score	0.79 (0.35)	−1.01 (0.32)	0.39 (0.31)	−0.02 (0.96)
INF-γ	0.00 (0.97)	−0.01 (0.76)	0.01 (0.30)	0.00 (0.69)
Age	−0.17 (0.45)	0.00 (0.99)	1.15 (<0.001)	0.67 (<0.001)
Sex (Male)	−2.20 (<0.001)	−3.58 (<0.001)	−1.26 (<0.001)	−0.59 (0.03)
SES	2.79 (<0.001)	4.90 (<0.001)	1.75 (<0.001)	0.94 (<0.01)
BMI Z-score	0.58 (0.42)	−0.39 (0.66)	0.34 (0.38)	0.01 (0.98)
TNF-α	0.04 (0.14)	0.06 (0.08)	0.00 (0.79)	0.00 (0.88)
Age	−0.18 (0.47)	−0.06 (0.85)	1.17 (<0.001	0.66 (<0.001)
Sex (Male)	−2.94 (.047)	−7.21 (<0.001)	−2.39 (0.001)	−1.22 (0.02)
SES	2.31 (0.02)	5.03 (<0.001)	1.78 (<0.001)	0.96 (<0.01)
BMI Z-score	0.80 (0.34)	−0.98 (0.33)	0.38 (0.33)	0.01 (0.96)

WRAML = Wide Range Assessment of Memory and Learning; PNIT = Philippines Non-Verbal Intelligence Test.

## Discussion

This study presents strong evidence supporting the existence of a relationship between NIDA and performance on verbal memory and a test that captures abstract thinking after controlling for important confounding variables. To our knowledge, this is the first study to both investigate and demonstrate an association between NIDA and cognitive function. Other work with this cohort had established that the main cause of NIDA in this population is AI, rather than hemolysis or other micro-nutrient deficiencies.[5],[6]. Implicating AI as a cause of cognitive deficits heightens its public health importance given its association with many infectious and non-infectious diseases of developing countries including HIV [32],[33] and malaria [34].

In addition to our results for NIDA, we also provide evidence of a significant association between IDA and performance on an intelligence test after rigorously adjusting for potential confounders. Though IDA was associated with lower scores on all four of the cognition tests before adjusting for confounders, the relationship between IDA and cognition remained for just one of the four tests after controlling for confounders, highlighting the correlations among risk factors for both iron deficiency and cognitive function and the potential for confounding without adjustment [35].

Studies examining cognitive deficits in the context of other exposures have found similar differences in outcomes for the tests we used. Compared to a group of children with no anemia, we found differences in the WRAML verbal memory index of approximately 4 points on a scaled score. To place this in perspective, children with attention deficit hyperactivity disorder (ADHD) had verbal memory index scores that were 6.8 points lower than children without ADHD [36]. Among Filipino children who were normal birthweght and breast fed for 12–18 months versus less than six months, adjusted scores on the PNIT were 1.6 points higher at age 8.5 [37].

In our previous work, *S. japonicum* infection, which has been demonstrated to cause NIDA, was related to decreased performance on the learning domain of the WRAML [18], whereas NIDA was not related to decreased learning performance scores. Thus *S. japonicum*'s effect on learning may occur through mechanisms other than NIDA. We also evaluated the role of *A. lumbricoides* because of its relationship to the WRAML verbal memory index, however, *A. lumbricoides* neither confounded nor acted as the distal determinant in a causal pathway linking this helminth to NIDA and ultimately decreased cognitive performance, suggesting other mechanisms are likely.

Inflammatory cytokines are involved in the development of AI. These cytokines, particularly TNF-α, IFN-γ and IL-6, are also known to induce a syndrome termed “sickness behavior,” characterized by fatigue, impaired sleep, and cognitive dysfunction [31],[38]. Most studies suggest these cytokines exert these effects by entering the brain through the blood-brain barrier and modifying inflammatory responses [39],[40]. Given the possibility that these cytokines could mediate the cognitive deficits in the context of AI through direct effects on the CNS, we evaluated their independent relationship with cognitive domains. The absence of a relationship between pro-inflammatory cytokines and cognition suggests that they are unlikely to be the major factor mediating cognitive impairment in this setting. Thus, it seems that AI-related decreases in iron bio-availability to the CNS, are the primary cause of cognitive impairment in this setting.

Based on both human and animal models, it is well accepted that IDA is related to cognitive impairment, most likely due to decreases in brain iron content. Iron is actively transported into the brain by transferrin receptors and is used for myelination and neurotransmitter synthesis. Iron is also necessary for brain-energy metabolism [41]. In this cohort, it is likely that iron's effect on neurotransmitter synthesis is of greater importance given most myelination occurs during late gestation until 24 months of age. We examined whether children who had normal total body levels of iron, but evidence of decreased iron transport to, and utilization by, host tissues as evidenced by anemia, might not perform as well on tests of cognitive function due to CNS iron deficiency. It is not surprising that, in the context of AI, where iron delivery to host tissues is already sufficiently decreased as to cause anemia, that other host tissues that are dependent on iron would be affected. Also in support of this conclusion is the fact that the same domains of cognitive function were adversely affected by NIDA and IDA, as one would expect if the proximal cause of lower performance is decreased delivery of iron to the CNS. Of note, memory function, as captured by the WRAML verbal memory index, has been demonstrated in many studies to be sensitive to iron status [10],[11].

Study limitations include the cross-sectional design limiting causal inferences and the possibility for residual confounding based on measured and unmeasured covariates. It is possible that children with AI had other infectious or non-infectious diseases that might be related both to AI and cognitive impairment. This is unlikely given children were screened for the presence of significant diseases. Further, the prevalence of HIV in this community is extremely low [42] and malaria is not endemic. S. *japonicum* infection was not related to the same cognitive deficits as AI. It is possible that there is variability in immune responses to S. *japonicum* and the many parasitic and other infectious diseases in this setting, such that children with more exuberant pro-inflammatory responses may experience greater AI, which may not be simply related to intensity of infection. It is also possible that our cognition tests, such as the WRAML, may not capture learning and memory abilities in the same manner as when used in US populations. This would likely lead to measurement error, however, rather than introduce bias. In addition, it is likely that some of our subjects actually had both iron deficiency anemia and AI, with some overlap in definitions possible. Current definitions preclude allowing for this combination, though it likely occurs frequently in developing countries. Further, it is possible that the group with NIDA had other causes of anemia, other than AI. We evaluated many other potential causes of NIDA, but cannot rule out other causes including genetic disorders such as thalassemias and G6PD deficiency or vitamin A deficiency. Though these entities may cause NIDA, there are less established mechanisms through which they would lead to cognitive impairment, as opposed to through alterations in iron metabolism in the context of AI as proposed.

Though IDA and AI may both cause cognitive deficits through decreased iron bio-availability to the CNS, the etiology and treatment differ substantially. Iron supplementation is generally provided for treatment of IDA. Iron supplementation in the context of AI, however, has minimal if any benefit, given AI leads to decreased iron absorption and movement of iron into storage forms. The costs and benefits of iron supplementation must be carefully weighed in this context, particularly given recent concerns that it may increase risk of malaria morbidity [43],[44]. The therapeutic approach of choice for AI is treatment of the underlying condition [1].

This study suggests that NIDA, largely due to AI, is associated with cognitive deficits in children. AI, caused by many diseases of lesser-developed countries, may further limit children's ability to take advantage of limited educational opportunities, and can only be addressed by treatment of underlying diseases.
